# Autologous hematopoietic stem cell transplantation in systemic sclerosis induces long-lasting changes in B cell homeostasis toward an anti-inflammatory B cell cytokine pattern

**DOI:** 10.1186/s13075-019-1889-8

**Published:** 2019-04-29

**Authors:** Michael Gernert, Hans-Peter Tony, Eva Christina Schwaneck, Ottar Gadeholt, Marc Schmalzing

**Affiliations:** Department of Medicine II, Rheumatology and Clinical Immunology, University of Würzburg, Oberdürrbacher Str. 6, 97080 Würzburg, Germany

**Keywords:** Systemic sclerosis, B cells, Memory B cells, naïve B cells, Autologous hematopoietic stem cell transplantation, Interleukin-10

## Abstract

**Background:**

Autologous hematopoietic stem cell transplantation (aHSCT) is performed in patients with aggressive forms of systemic sclerosis (SSc). The profile of B cell reconstitution after aHSCT is not fully understood. The aim of this study was to investigate changes of B cell subsets and cytokine production of B cells in patients with SSc after aHSCT.

**Methods:**

Peripheral blood of six patients with SSc was collected at defined intervals up to 16 months after aHSCT. Immunophenotyping was performed, and B cell function was determined by measuring cytokine secretion in supernatants of stimulated B cell cultures.

**Results:**

Within 1 month after aHSCT, a peak in the percentage of CD38^++^/CD10^+^/IgD^+^ transitional B cells and CD38^++^/CD27^++^/IgD^−^ plasmablasts was detected. Long-term changes persisted up to 14 months after aHSCT and showed an increased percentage of total B cells; the absolute B cell number did not change significantly. Within the B cell compartment, an increased CD27/IgD^+^ naïve B cell percentage was found whereas decreased percentages of CD27^+^/IgD^+^ pre-switched memory, CD27^+^/IgD^−^ post-switched memory, and CD27^−^/IgD^−^ double-negative B cells were seen after aHSCT. Cytokine secretion in B cell cultures showed significantly increased IL-10 concentrations 13 to 16 months after aHSCT.

**Conclusion:**

A changed composition of the B cell compartment is present for up to 14 months after aHSCT indicating positive persisting effects of aHSCT on B cell homeostasis. The cytokine secretion profile of B cells changes in the long term and shows an increased production of the immune regulatory cytokine IL-10 after aHSCT. These findings might promote the clinical improvements after aHSCT in SSc patients.

**Electronic supplementary material:**

The online version of this article (10.1186/s13075-019-1889-8) contains supplementary material, which is available to authorized users.

## Background

Systemic sclerosis (SSc) is a heterogeneous and devastating autoimmune disease characterized by inflammation, vasculopathy, and fibrosis. Fibrosis occurs in the skin and the internal organs and is often refractory to standard immunosuppressive therapy [1]. Depending on the extent of skin involvement, SSc can be divided into a limited cutaneous form (lcSSc) or a diffuse cutaneous form (dcSSc) [2]. SSc is the rheumatic disease with the highest case mortality, mainly driven by pulmonary hypertension and lung fibrosis [3]. The use of immunosuppressive therapy is restricted to patients with diffuse progressive skin disease and progressive inner organ involvement mainly of the lungs, whereas vasculopathy can hardly be controlled by immunosuppressive therapy.

So far, studies have not been able to show that conventional and biologic disease-modifying antirheumatic drugs (DMARDs) have more than a limited effect on disease manifestations; however, there is some evidence for the efficacy of mycophenolate mofetil and cyclophosphamide [4, 5]. Case series suggest a slight benefit of rituximab to skin and lung involvement [6, 7]. A phase 3-study investigated the efficacy of tocilizumab in SSc patients and indicates positive effects on lung fibrosis [8].

Of all treatment options for SSc autologous hematopoietic stem cell transplantation (aHSCT) seems to be the most efficacious therapy, but treatment toxicity and treatment-related mortality have to be considered in this population with limited organ function. Three randomized trials (one unicenter, two multicenter) have shown superior efficacy concerning skin and lung involvement in patients with diffuse cutaneous SSc compared to treatment with intravenous cyclophosphamide once per month for 6 months [9] or intravenous cyclophosphamide once per month for 12 months [10, 11]. The two multicenter trials also have shown a long-term survival benefit, although early treatment-related mortality was increased [10, 11]. For aHSCT, patients’ stem cells are mobilized using chemotherapy and granulocyte colony-stimulating factor (G-CSF). These stem cells were enriched by CD34^+^-selection and were reinfused after an immunoablative conditioning regimen consisting of cyclophosphamide and anti-thymocyte globulin (ATG) in most cases. Nevertheless, relapses and disease progression can occur also after aHSCT, and so far, there are no studies that have examined treatment options after aHSCT and few data is available about how aHSCT evokes its effects.

The pathogenesis of SSc has been widely investigated. Interstitial fibrosis is the major mechanism causing organ damage. This fibrosis seems to be promoted by disturbed T cell subsets [12, 13] and by secretion of pro-fibrotic cytokines from CD8^+^ T cells [14]. Vasculopathy is correlated with specialized circulating angiogenic T cells [15]. Furthermore, an altered B cell system has been described demonstrating that B cells play an important role in the pathogenesis of SSc [16]: Autoimmune properties of B cells are increased in SSc patients by an overexpression of CD19 [17] and B cell activating factor (BAFF) [18]. Plasma cells of SSc patients produce anti-topoisomerase 1/Scl70 auto-antibodies, which are associated with a more extensive skin involvement [19], and alteration of B cell subsets in SSc patients are described [20]. Disturbed cytokine profiles in SSc patients are important in creating a pro-fibrotic state [21].

An important B cell subset in autoimmune diseases is the CD19^+^/CD27^−^/IgD^−^ (double-negative) B cell subset. Although they do not express CD27, double-negative B cells seem to belong to the memory compartment, as their immunoglobulin receptors show somatic hypermutations [22]. In rheumatoid arthritis (RA) and systemic lupus erythematosus (SLE), double-negative B cells are increased [23, 24]. In concordance with that, higher numbers of double-negative B cells correlate with a better response to rituximab in RA patients [25].

Interleukin (IL)-10 is a cytokine of special interest for the investigation of the pathogenesis of SSc. IL-10 terminates inflammatory processes and stimulates growth and differentiation of various cell types including B cells, T cells, NK cells, dendritic cells, and endothelial cells among others [26]. Downstream the IL-10 receptor, the signal transducer and activator of transcription 3 (STAT3) pathway and the phosphoinositide 3-kinase (PI3K) pathway are activated, resulting in the inhibition of antigen presentation and the inhibition of secretion of pro-inflammatory cytokines mainly in macrophages and dendritic cells [27]. That is why special attention was turned to IL-10-producing B cells (Bregs) which can be located within the CD19^+^/CD24^high^/CD38^high^ or CD19^+^/CD24^high^/CD27^+^ subpopulation [28]. A reduction of Bregs [29] and a reduced ability to produce IL-10 is described in SSc patients [30].

The aim of the present study was to elucidate the changes in B cell subsets and in the B cell function in SSc patients after receiving aHSCT. Those insights might indicate treatment targets for SSc patients with progressive or relapsing disease after aHSCT.

## Patients and methods

### Patients

Six patients (five female, one male; mean age 44.2 years, range from 23 to 58 years) who met the ACR/EULAR criteria [31] for SSc were included in the study. At the time of aHSCT, five patients had a diffuse cutaneous form (dsSSc) and one patient a limited cutaneous form (lcSSc), two patients showed positivity for Scl70-antibodies, and four patients had pulmonary fibrosis due to SSc. Patients’ characteristics are summarized in Table 1.

**Table 1:** Characteristics of the study population*

Patient	Sex	Age at aHSCT	Cutaneous form	Scl-70 positivity	Pulmonary fibrosis	Troponin positivity	CD34^+^-selected graft
1	F	38	dc	+	+	–	+
2	F	53	lc	–	–	+	+
3	M	58	dc	–	+	+	–
4	F	47	dc	–	+	–	+
5	F	23	dc	+	–	+	–
6	F	46	dc	–	+	–	+

*F female, *M* male, *dc* diffuse cutaneous form, *lc* limited cutaneous form

We performed a prospective, non-interventional cohort study, which was approved by the local ethics committee. All patients gave their written informed consent. Participation in this study had no influence on their therapy.

### Myeloablative autologous hematopoietic stem cell transplantation

All patients suffered from progressive disease under conventional immunosuppressive treatment and therefore underwent aHSCT in the years 2014 to 2017. They received a treatment protocol analogous to that used in the ASTIS trial [10]: Patients received 2 g/m^2^ cyclophosphamide for mobilization of autologous hematopoietic stem cells together with a minimum daily dose of 105 μg granulocyte colony-stimulating factor (G-CSF), starting on day 2 after cyclophosphamide, followed by leukapheresis. The autologous hematopoietic stem cells underwent CD34^+^-selection using immunomagnetic separation (CliniMACS CD34 Complete Kit, Miltenyi Biotec, Bergisch Gladbach, Germany) in four out of six patients. In two patients, stem cells were not CD34^+^-selected due to low stem cell numbers in the leukapheresis product. Then, patients obtained an immunoablative conditioning regimen containing a total of 200 mg/kg cyclophosphamide (on days 1–4) plus 30 mg/kg rabbit ATG (on days 2–5). The autologous hematopoietic stem cells (minimum dose of 2.0 × 10^6^ CD34^+^ cells per kilogram body weight) were then reinfused (on day 6).

### Immunophenotyping

Peripheral blood was obtained before (range 5 to 12 weeks) and after (at month 1, 2, 3, 5–7, and 12–14) aHSCT. Distribution of B cell subsets was obtained from fresh blood via immunophenotyping. Staining was performed with the Navios cytometer (Beckman Coulter, Krefeld, Germany) using the following antibodies: CD19-phycoerythrin-cyanin (PC) 7, CD20-allophycocyanin (APC) 750, CD45-Krome Orange, CD27-phycoerythrin-Texas Red-X (ECD), CD38-PC5.5 (each Beckman Coulter, Krefeld, Germany), IgD-fluorescein isothiocyanate (FITC), CD10-phycoerythrin (PE) (each BD Biosciences, San Jose, CA), CD21-Pacific Blue (Exbio, Prague, Czech Republic), and IgM-APC (BioLegend, San Diego, CA). Lymphocytes were identified by using forward versus sideward scatter. Within the lymphocyte gate, at least 3000 events were collected and CD19^+^ cells were identified as B cells. Transitional B cells were defined as CD38^++^/CD10^+^/IgD^+^, pre-switched memory B cells as CD27^+^/IgD^+^, post-switched memory B cells as CD27^+^/IgD^−^, double-negative B cells as CD27^−^/IgD^−^, naïve B cells as CD27^-^/IgD^+^, and circulating plasmablasts as CD38^++^/CD27^++^/IgD^−^.

### Preparation of peripheral blood mononuclear cells (PBMCs) and B cell enrichment

Peripheral blood in EDTA-tubes (15–20 ml) was obtained before (range 6 to 12 weeks) and after (range 13 to 16 months) aHSCT and processed with Ficoll-Paque Plus separation (GE Healthcare, Munich, Germany) according to the manufacturer’s instructions to receive PBMCs. PBMCs were stored in liquid nitrogen before they were incubated with CD19 monoclonal antibody-coupled microbeads to separate B cells by magnetic cell sorting (MACS; Miltenyi Biotec, Bergisch Gladbach, Germany). Each PBMC sample was loaded onto two MACS columns successively to achieve a B cell purity over 95%.

### B cells cultures and cytokine measuring

The enriched B cells were incubated over 24 h with 10 μg/ml cytosine guanine dinucleotide (CpG ODN 2006, InvivoGen, Toulouse, France) in 96-well plates at a concentration of 0.5–1 × 10^6^ cells/ml. Supernatants were then collected and stored at − 80 °C. Cytokines from supernatants of the B cell cultures were measured using cytometric bead array (CBA flex set; BD bioscience, San Jose, CA) in a LSR II cytometer (BD bioscience, San Jose, CA). Concentrations of cytokines were calculated using FCAP array software (BD bioscience, San Jose, CA).

### Statistical analysis

Samples were tested for normal distribution by performing Shapiro-Wilk tests and Q-Q plots. If normal distribution was determined, means ± standard deviations (SD) were calculated and differences were analyzed using a two-tailed paired *t* test. If normal distribution could not be determined, medians with interquartile ranges (IQR) were calculated and Wilcoxon signed-rank tests were performed to detect differences between groups. SPSS Statistics v 25.0 (IBM, Armonk, NY) and Excel (Microsoft, Redmond, WA) were used. Differences were considered significant when *P* values were less than 0.05.

## Results

### Increased percentage of total B cells after aHSCT

**Fig. 1:**
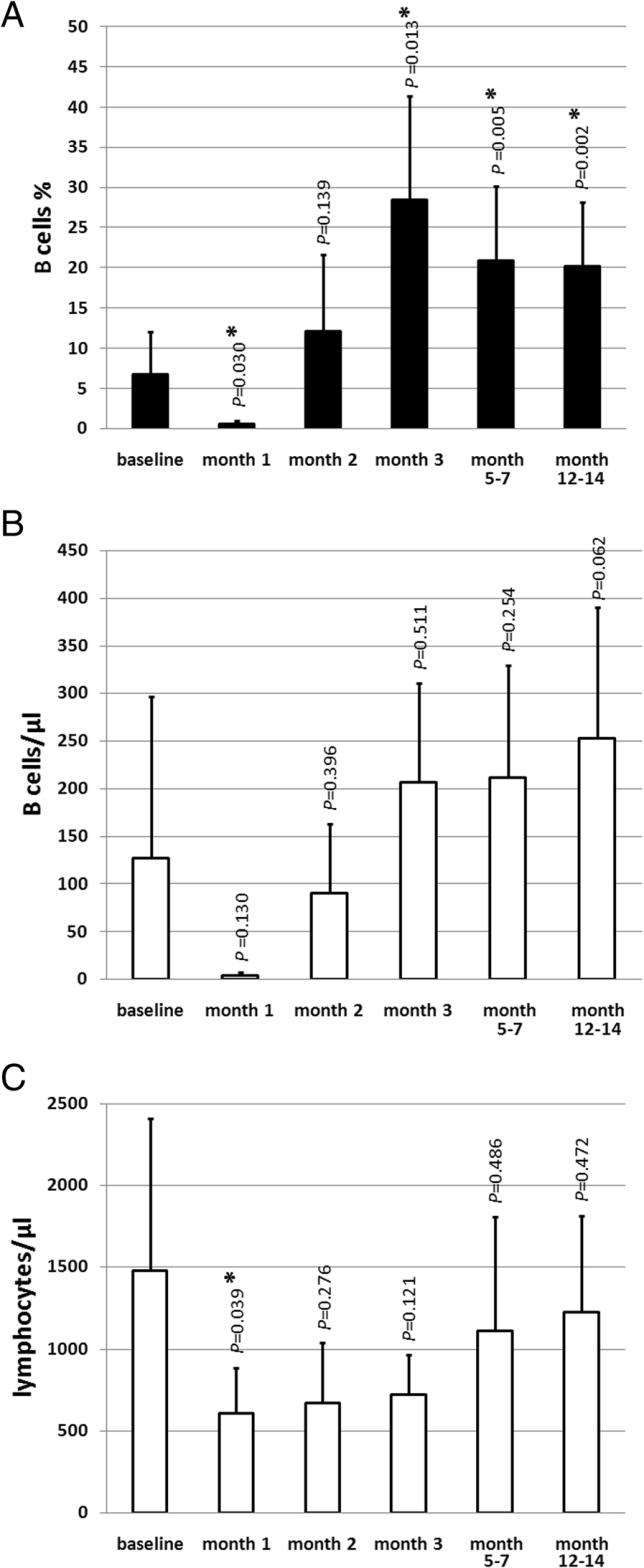
Changes of B cell percentages (**a**), B cell numbers (**b**), and lymphocyte numbers (**c**) after aHSCT. **a** B cell percentages within the lymphocyte gate at different time points. Baseline (before aHSCT), month 1, month 2, month 3, months 5–7, and months 12–14 after aHSCT. **b** Corresponding B cell numbers/μl and **c** lymphocyte numbers/μl at respective time points. *n* = 6 (except months 2 and 3 *n* = 5); mean ± SD; *significant (*P* < 0.05) difference compared to baseline in the two-tailed paired *t* test

In the first month after aHSCT, the percentage of total B cells within the lymphocyte gate showed the lowest values (0.6 ± 0.5%; mean ± SD) compared to the baseline values before aHSCT (6.8 ± 5.3%; *P* = 0.030). In that stage of the lymphocyte repopulation, the predominant population were NK cells (data not shown). Total B cell percentages showed the highest values 20.7 weeks (range 10–47 weeks) after aHSCT. Total B cell percentages increased significantly 4.2-fold from 6.8 ± 5.3% at baseline to 28.5 ± 12.9% 3 months after aHSCT (*P* = 0.013). A significant increase in total B cell percentages was maintained until 14 months after aHSCT (Fig. 1a). The longest follow-up in one patient comprised 2 years and showed a sustained 4.1-fold increase of the total B cell percentage (4.8% at baseline compared to 19.5% 2 years after aHSCT).

Absolute numbers of B cells had the lowest values 1 month after aHSCT and increased continuously over time, starting from the second month after aHSCT, without showing any significant changes compared to baseline (Fig. 1b). Lymphocyte numbers (Fig. 1c) showed a mean of 1478.3 ± 929.9/μl at baseline. The minimum of lymphocyte numbers was detected 1 month after aHSCT (605.0 ± 277.8/μl; *P* = 0.039). From month two after aHSCT, lymphocyte numbers recovered and did not show significant differences to baseline.

### Early phase of B cell repopulation

**Fig. 2:**
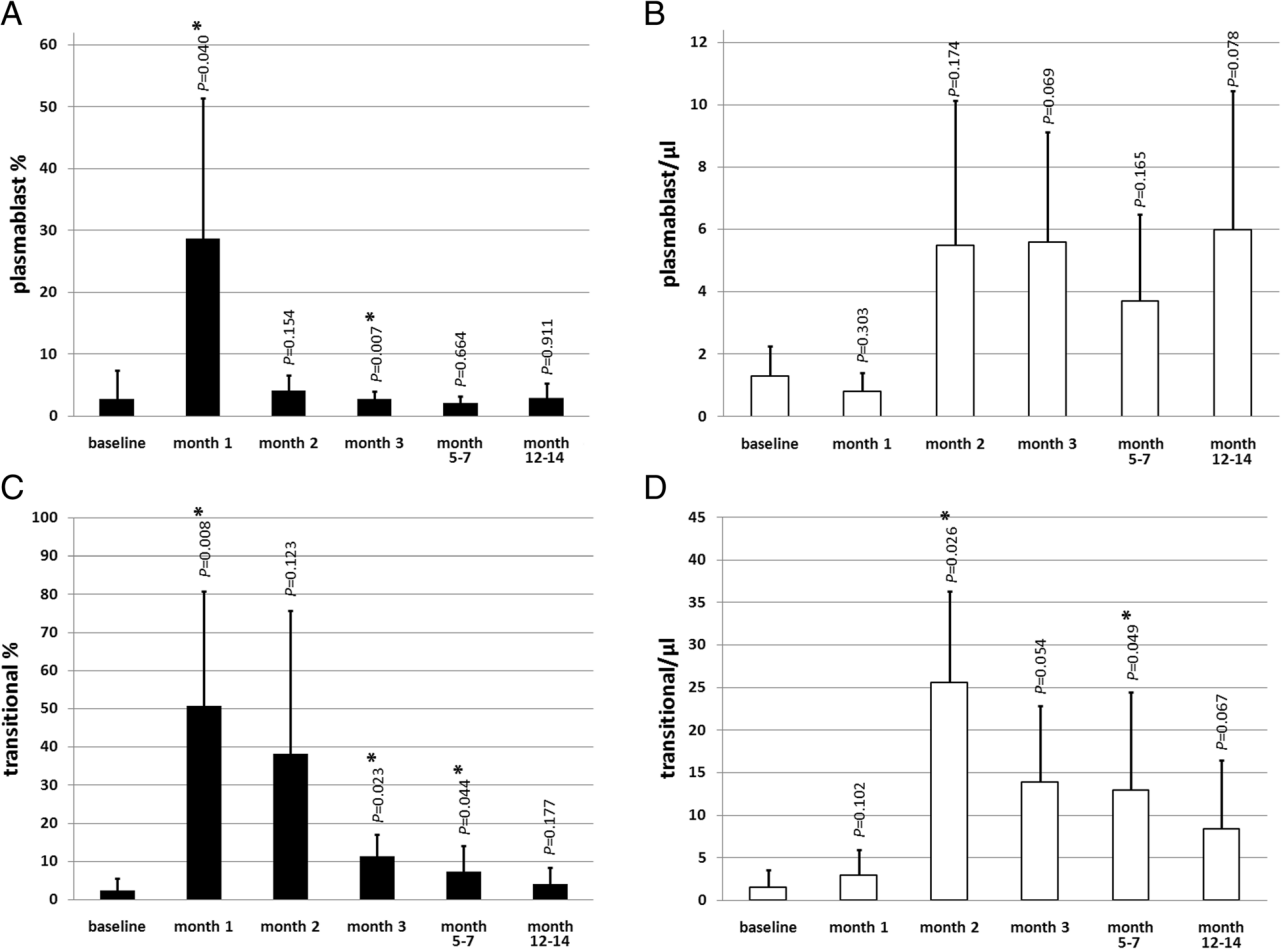
Changes of plasmablast (CD38^++^/CD27^++^/IgD^−^) percentages (**a**), plasmablast numbers/μl (**b**), transitional B cell (CD38^++^/CD10^+^/IgD^+^) percentages (**c**), and transitional B cell numbers/μl (**d**) after aHSCT at respective time points. *n* = 6 (except month 2 *n* = 4 and month 3 *n* = 5); mean ± SD; *significant (*P* < 0.05) difference compared to baseline in the two-tailed paired *t* test

The subset analysis within the B cell compartment revealed a specific repopulation pattern after aHSCT. In the first month after aHSCT, a 10-fold increase could be detected in the percentage of plasmablasts (CD38^++^/CD27^++^/IgD^−^) from 2.8 ± 4.6% at baseline compared to 28.7 ± 22.7% 1 month after aHSCT (*P* = 0.040; Fig. 2a). The absolute numbers of plasmablasts did not change significantly after aHSCT (Fig. 2b). A 21-fold increase in the percentage of transitional B cells (CD38^++^/CD10^+^/IgD^+^) from 2.4 ± 3.2% at baseline compared to 50.8 ± 29.9% 1 month after aHSCT (*P* = 0.008) was present (Fig. 2c). Absolute numbers of transitional B cells showed a peak of 25.6 ± 10.7/μl 2 months after aHSCT compared to baseline 1.6 ± 2.0/μl (*P* = 0.026; Fig. 2d). Those changes diminished over time, with no significant differences in plasmablast and transitional B cell percentages and absolute numbers 1 year after aHSCT compared to baseline.

### Long-term changes in B cell subsets after aHSCT

**Fig. 3:**
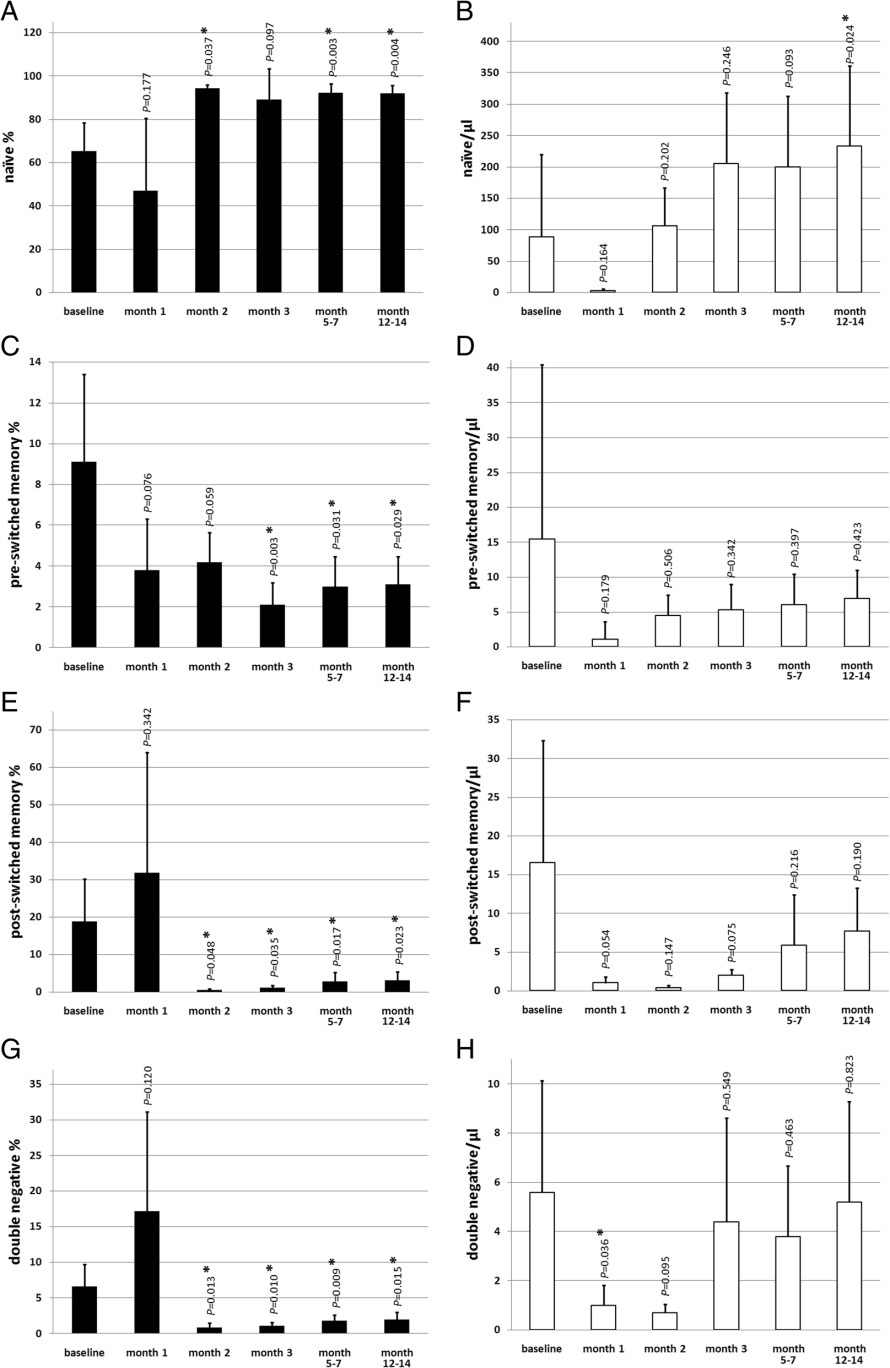
Naïve B cell (CD27^-^/IgD^+^) percentages (**a**) and naïve B cell numbers/μl (**b**), pre-switched memory B cells (CD27^+^/IgD^+^) percentages (**c**) and pre-switched memory B cell numbers/μl (**d**), post-switched memory B cell (CD27^+^/IgD^−^) percentages (**e**) and post-switched memory B cell numbers/μl (**f**), double-negative B cell (CD27^−^/IgD^−^) percentages (**g**) and double-negative B cell numbers/μl (**h**), at baseline and after aHSCT at respective time points. *n* = 6 (except month 2 *n* = 4 and month 3 *n* = 5); mean ± SD; *significant (*P* < 0.05) difference compared to baseline in the two-tailed paired *t* test

Starting from 2 months after aHSCT, distinct changes in the B cell subsets could be detected. The B cell repopulation was mainly accomplished by naïve B cells (CD27^−^/IgD^+^). Naïve B cell percentages increased significantly 1.4-fold from 65.4 ± 13.2% at baseline to 91.9 ± 3.9% 1 year after aHSCT (*P* = 0.004; Fig. 3a). Also, absolute naïve B cell numbers showed a significant increase from 88.9 ± 131.4/μl at baseline to 233.6 ± 127.7/μl 1 year after aHSCT (*P* = 0.024; Fig. 3b).The memory B cell compartment (CD27^+^) did not recover until 1 year after aHSCT. The pre-switched memory B cell percentages (CD27^+^/IgD^+^) declined from 9.1 ± 4.3% at baseline to 3.1 ± 1.4% 1 year after aHCT (*P* = 0.029; Fig. 3c). Similar changes could be detected in the post-switched memory B cells (CD27^+^/IgD^−^): Percentages declined from 18.9 ± 11.4% at baseline to 3.1 ± 2.4% 1 year after aHSCT (*P* = 0.023; Fig. 3e). Absolute numbers of pre-switched (Fig. 3d) or post-switched memory B cells (Fig. 3 f) did not change significantly after aHSCT. Double-negative B cell percentages (CD27^−^/IgD^−^) showed a constant decrease after aHSCT (6.6 ± 3.2% at baseline versus 2.0 ± 1.0% 1 year after aHSCT; *P* = 0.015; Fig. 3g), whereas absolute numbers of double-negative B cells showed no long-term changes (Fig. 3h).

### Increased IL-10 secretion by B cells after aHSCT

**Fig. 4:**
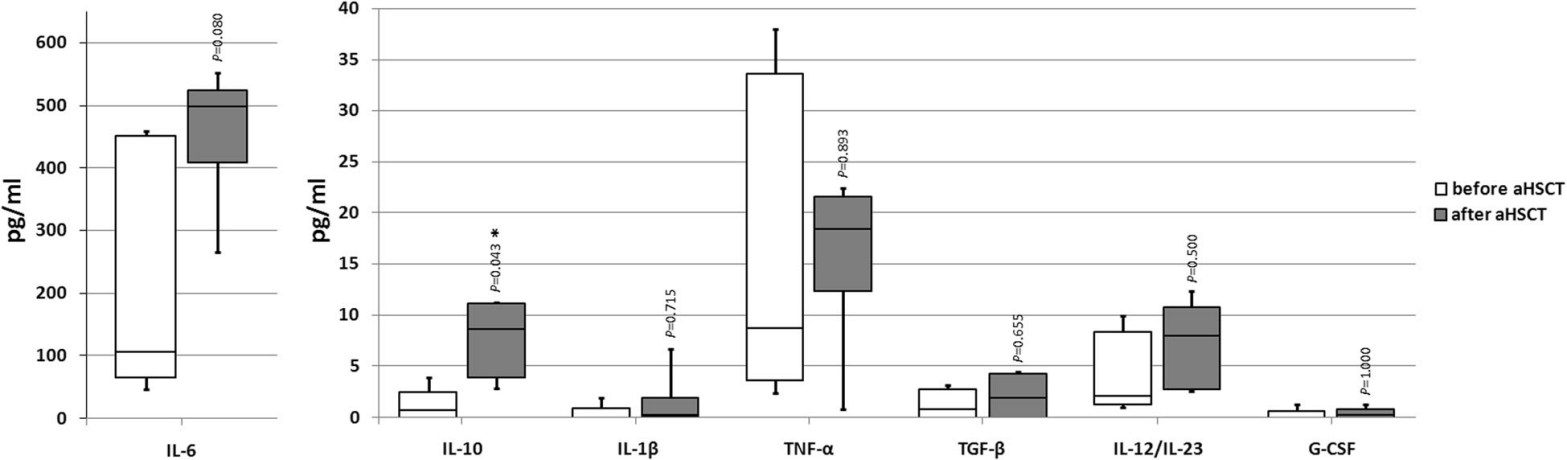
Cytokine concentrations in CpG-stimulated B cell cultures (B cell purity over 95%) from SSc patients before (white boxes; *n* = 5) and 11–16 months after aHSCT (gray boxes; *n* = 6). Boxes show interquartile range and median, whiskers show the lowest or highest value; *P < 0.05 using the Wilcoxon signed-rank test

To evaluate the influence of aHSCT on the B cell function of SSc patients, B cell cultures were stimulated with CpG ODN and cytokines were measured in the supernatants. The effect of CpG ODN stimulation on the cytokine secretion is described in Additional file 1: Figure S1. B cells after aHSCT showed a higher secretion of IL-10 compared to the levels before transplantation. The median concentration of IL-10 before aHSCT was 0.8 pg/ml (IQR 0.0–2.5 pg/ml) versus 8.6 pg/ml (IQR 3.9–11.2 pg/ml) after aHSCT (*P* = 0.043). No significant changes were observed in the secretion of the following cytokines after aHSCT: IL-6, IL1-β, TNF-α, TGF-β, IL-12/IL-23, and G-CSF (Fig. 4). No secretion of IL-1-α could be detected (data not shown).

### Improved skin involvement and stabilized lung function of SSc patients after aHSCT

Clinical data was assessed before and 10 to 18 months after aHSCT. The mean modified Rodnan skin score (mRSS) of the six SSc patients improved after aHSCT. Before aHSCT, the mean mRSS was 21.8 ± 11.3 (mean mRSS ± SD; range 9–41); after aHSCT, the mean mRSS was 11.5 ± 7.3 (range 2–20; *P* = 0.005). The mean diffusing capacity for carbon monoxide (DLCO) showed stable values, which means no worsening of lung function. Before aHSCT, DLCO was 45.3 ± 20.7% (mean % of predicted DLCO ± SD), and after aHSCT, it was 47.5 ± 23.8% (*P* = 0.315). The mean forced vital capacity (FVC) was stable over the course of the study. Before aHSCT, it was 80.0 ± 19.1% (mean % of predicted FVC ± SD), and after aHSCT, it was 88.3 ± 24.0% (*P* = 0.066). Morphological pulmonary fibrosis, documented as interstitial lung disease by thoracic computed tomography, showed stable findings or even a decline in fibrotic manifestations after aHSCT.

## Discussion

This prospective study analyzed the composition of the peripheral B cell compartment and the cytokine profile of purified B cells upon CPG stimulation of SSc patients before and after aHSCT.

After aHSCT, an increased total B cell percentage within the lymphocytes was seen. Within the B cell compartment, increased naïve B cells, decreased memory B cells (pre- and post-switched), and decreased double-negative B cells could be detected. Those changes were long-term changes and stable at least until 1 year after aHSCT.

Similar repopulation patterns after aHSCT have been reported in other autoimmune diseases, for example SLE or multiple sclerosis with an incline in naïve B cell frequencies and a decline in memory B cells [32, 33]. Also, in hematologic malignancies, an increased naïve B cell compartment after aHSCT was described [34].

The B cell repopulation pattern after aHSCT described is similar to the repopulation after B cell depleting treatment with rituximab. Application of rituximab caused a long-lasting reduction of memory B cells [35], persisting even after repeated treatment with rituximab in RA patients [36]. In contrast to the treatment with rituximab after aHSCT, the total B cell percentage is increased 1 year after treatment. An increased percentage of total B cells in absence of a change in the absolute numbers of total B cells after aHSCT could be explained by the previously described T cell lymphopenia, especially CD4^+^ T cell lymphopenia, following aHSCT [37].

Our six patients achieved an improved mRSS and a stabilized lung function after aHSCT, similar to other cohorts [10]. To mediate these clinical improvements, the aHSCT aims to accomplish a reset of the immune system and of the B cell system [38]. A dysfunctional B cell homeostasis is postulated to play an important role in the pathogenesis of SSc [16]. aHSCT might evoke a shift of the B cell homeostasis toward a regulatory phenotype. As seen in this study, the secretion of the immune regulatory cytokine IL-10 is significantly increased after aHSCT. This could be explained by the recurrence of regulatory B cells (Bregs) after aHSCT as Bregs are often defined through their ability to produce IL-10 and are known to be an important B cell population producing IL-10 [39]. As we measured the IL-10 production in cultures of total B cells, we cannot distinguish which B cell subset mainly produced the detected IL-10. IL-10 might not only originate from Bregs. As naïve B cells are described to be important producers of IL-10 [40], and we could show increased percentages and absolute numbers after aHSCT, naïve B cells might have contributed to a high extend to the IL-10 production. Therefore, the increased IL-10 concentration might be a reflection of the regenerating B cell system. The production of IL-10 is not restricted to B cells, but B cell-derived IL-10 seems to be very important in the pathogenesis of SSc. This could be shown in a bleomycin-induced mouse model. Mice with a B cell-specific deficiency in the IL-10 production showed a more severe skin and lung fibrosis, compared to mice with sustained IL-10 production by B cells [41].

Administration of IL-10 to treat autoimmune diseases has been studied extensively and led to a reduction of psoriatic plaques in psoriasis and ameliorated the disease activity in intestinal bowel disease [27, 42]. In contrast to that, blocking IL-10 showed a significant clinical improvement in SLE patients in a small open-label study [43]. Therefore, using IL-10 as a therapeutic agent seems to be ambivalent due to its pleiotropic effects in different autoimmune diseases.

SSc patients can show progressive disease manifestations after aHSCT. Plasma cells can survive in bone marrow niches despite cytoreductive or immunosuppressive therapies [44], which could explain refractory disease or relapse [45, 46] and could be one reason why even after aHSCT further treatment is necessary. Data for treatment after aHSCT in SSc patients is limited. As an increased B cell percentage after aHSCT is present, one could speculate that the application of rituximab could be an effective therapy. In two of our patients, rituximab was used successfully without adverse effects after aHSCT due to progressive skin involvement. Application of rituximab already has shown an improvement in the mRSS and stabilized the lung function [6] or even improved the lung function [7] of SSc patients.

Limitations of our study arise due to the relatively small number of included patients. Further investigations of SSc patients after aHSCT are necessary to support our findings.

## Conclusion

In summary, aHSCT causes distinct changes in the B cell compartment with an increased B cell percentage and a decreased memory B cell compartment. The predominant B cell subpopulation after aHSCT is naïve B cells. After aHSCT, B cells show an increased secretion of the immune regulatory cytokine IL-10. All of these changes could play a role in the persisting clinical improvements of SSc patients after aHSCT. Elevated total B cell percentages and changes in cytokine concentrations offer possibilities for future treatments of progressive SSc after aHSCT.

## Additional file

Additional file 1: **Figure S1.** Toll-like receptor 9 stimulator CpG ODN induces cytokine secretion in B cell cultures of SSc patients. B cell cultures (two samples before aHSCT and four samples after aHSCT) from SSc patients who were not treated with CpG (no CpG; white boxes) or were treated with CpG (10 μg/ml; gray boxes). The cytokine concentrations in the supernatants were measured. A significant increase upon CpG ODN stimulation could be detected for IL-6 (20.0 pg/ml [IQR 13.3–30.3 pg/ml] versus 498.3 pg/ml [IQR 364.5–554.4 pg/ml]; *P* = 0.028), for IL-10 (0.0 pg/ml [IQR 0.0–0.2 pg/ml] versus 8.5 pg/ml [IQR 0.6–9.5 pg/ml]; *P* = 0.043) and for TNF-α (0.0 pg/ml [IQR 0.0–0.2 pg/ml] versus 20.0 pg/ml [IQR 15.7–26.3 pg/ml]; *P* = 0.028) (median concentration without CpG ODN versus median concentration with CpG ODN). No differences were seen in the levels of IL-1-β, TGF-β, IL-12/IL-23, and G-CSF after CpG ODN stimulation. *n* = 6; boxes show interquartile range and median, whiskers show lowest or highest value; *P < 0.05 using the Wilcoxon signed-rank test. (TIF 2312 kb)

## Abbreviations

ACR: American College of Rheumatology
aHSCT: Autologous hematopoietic stem cell transplantation
ATG: Anti-thymocyte globulin
BAFF: B cell activating factor
Bregs: Regulatory B cells
CD: Cluster of differentiation
CpG ODN: Cytosine guanine dinucleotide
dcSSc: Diffuse cutaneous form of systemic sclerosis
DLCO: Diffusing capacity for carbon monoxide
DMARDs: Disease-modifying anti rheumatic drugs
EULAR: The European League Against Rheumatism
FVC: Forced vital capacity
G-CSF: Granulocyte colony-stimulating factor
IgD: Immunoglobulin D
IL: Interleukin
IQR: Interquartile range
lcSSc: Limited cutaneous form of systemic sclerosis
mRSS: Modified Rodnan skin score
PBMCs: Peripheral blood mononuclear cells
PI3K: Phosphoinositide 3-kinase
RA: Rheumatoid arthritis
SLE: Systemic lupus erythematosus
SSc: Systemic sclerosis
STAT3: Signal transducer and activator of transcription 3
TGF-β: Transforming growth factor-beta
TNF-α: Tumor necrosis factor-alpha
