# Supplementary figures and images for: Autologous hematopoietic stem cell transplantation in systemic sclerosis induces long-lasting changes in B cell homeostasis toward an anti-inflammatory B cell cytokine pattern
Source: Arthritis Res Ther. 2019 Apr 29;21:106. doi: 10.1186/s13075-019-1889-8 (PMC6489316; doi:10.1186/s13075-019-1889-8)

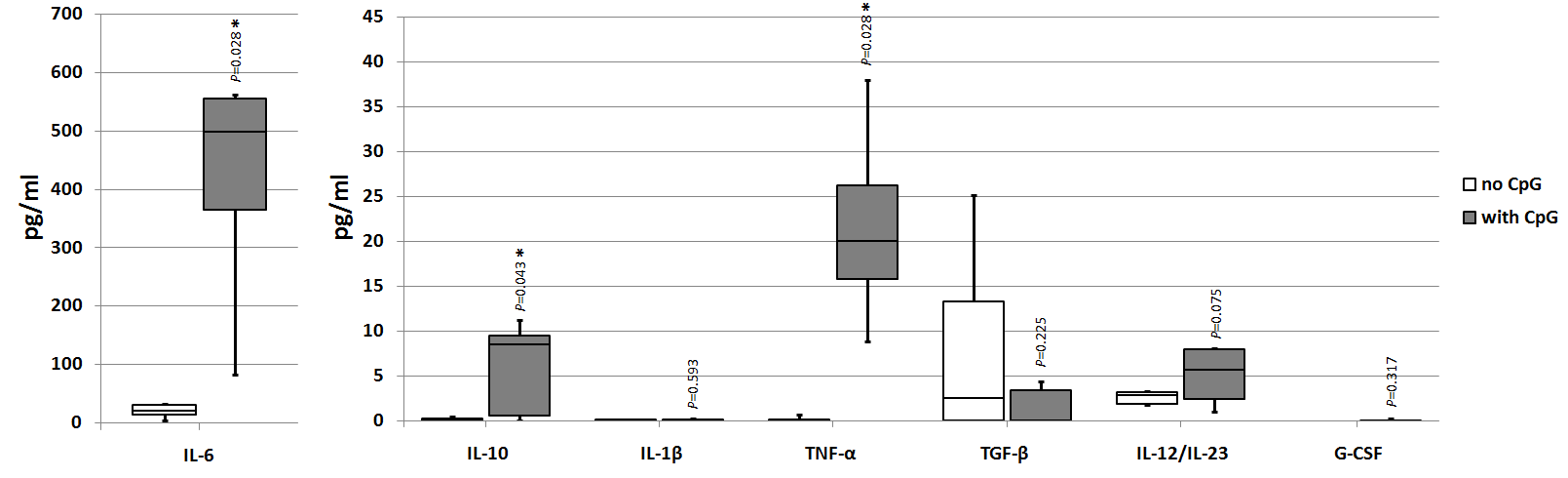

Supplement: Supplementary file 1 — Figure S1. Toll-like receptor 9 stimulator CpG ODN induces cytokine secretion in B cell cultures of SSc patients. B cell cultures (two samples before aHSCT and four samples after aHSCT) from SSc patients who were not treated with CpG (no CpG; white boxes) or were treated with CpG (10 μg/ml; gray boxes). The cytokine concentrations in the supernatants were measured. A significant increase upon CpG ODN stimulation could be detected for IL-6 (20.0 pg/ml [IQR 13.3–30.3 pg/ml] versus 498.3 pg/ml [IQR 364.5–554.4 pg/ml]; P = 0.028), for IL-10 (0.0 pg/ml [IQR 0.0–0.2 pg/ml] versus 8.5 pg/ml [IQR 0.6–9.5 pg/ml]; P = 0.043) and for TNF-α (0.0 pg/ml [IQR 0.0–0.2 pg/ml] versus 20.0 pg/ml [IQR 15.7–26.3 pg/ml]; P = 0.028) (median concentration without CpG ODN versus median concentration with CpG ODN). No differences were seen in the levels of IL-1-β, TGF-β, IL-12/IL-23, and G-CSF after CpG ODN stimulation. n = 6; boxes show interquartile range and median, whiskers show lowest or highest value; *P < 0.05 using the Wilcoxon signed-rank test. (TIF 2312 kb) [file 13075_2019_1889_MOESM1_ESM.tif]
